# Body Mass Index (BMI) Trajectories from Birth to 11.5 Years: Relation to Early Life Food Intake

**DOI:** 10.3390/nu4101382

**Published:** 2012-10-09

**Authors:** Frances L. Garden, Guy B. Marks, Judy M. Simpson, Karen L. Webb

**Affiliations:** 1 Sydney School of Public Health, The University of Sydney, Sydney, NSW 2006, Australia; Email: judy.simpson@sydney.edu.au; 2 Woolcock Institute of Medical Research, 431 Glebe Point Road, Glebe, NSW 2037, Australia; Email: guy.marks@sydney.edu.au; 3 Atkins Center for Weight and Health, University of California at Berkeley, 119 Morgan Hall, Berkeley, CA 94720, USA; Email: karenw05@berkeley.edu

**Keywords:** body mass index, developmental trajectory, obesity, breastfeeding, dietary intake, nutrition, diet, infant

## Abstract

Recent research has shown that the pattern of change over time, or trajectory, of body mass index (BMI) varies among children. However, the factors that underlie the heterogeneity in these trajectories remain largely unexplored. Our aim was to use a growth mixture model to empirically identify classes of BMI trajectories (from birth to 11.5 years) and examine the effects of breastfeeding, introduction of solids, as well as food and nutrient intake at 18 months on these BMI trajectories. We identified three BMI growth trajectories between birth and age 11.5 years, separately in boys and girls. Breastfeeding duration less than six months and the early introduction of solids did not adversely influence BMI trajectories in our sample but high intakes of meat, particularly high fat varieties, and high intakes of carbohydrate at age around 18 months were associated with a high BMI trajectory in boys. It is not clear whether these dietary factors confer a direct risk of higher BMI in childhood or are markers for other dietary patterns that are present early and/or develop through childhood and contribute to higher BMI.

## 1. Introduction

Recent research has shown that the pattern of change over time, or trajectory, of body mass index (BMI) varies among children [1,2,3,4,5,6]. However, the factors that underlie the heterogeneity in these trajectories remain largely unexplored. 

An understanding of the variation in the development of childhood overweight and obesity, and the factors contributing to it, is underpinned by appropriate longitudinal models for analysis of repeated BMI assessments. Statistical models for tracking BMI and weight status changes over time have utilized growth mixture modeling, a method that defines distinct groups, often called classes, within a sample based on their response patterns over time. Members of the same group (class) share a similar pattern of change over time. Each individual within the study sample is assigned a probability of belonging to each class. In some analyses, individuals are assigned to the class for which they have the highest probability of membership. Membership of a specific class can be used as an outcome variable or a predictor variable in subsequent analyses. The advantage of growth mixture modeling over traditional analyses of changes in weight over time using random effects models is that, unlike the latter, the former does not require an assumption that all members of the sample come from the same underlying distribution. Hence, growth mixture modeling is more flexible and robust than traditional methods of analyzing longitudinal data on weights and BMI.

In the Childhood Asthma Prevention Study (CAPS) we collected data, prospectively from birth, on height and weight, breastfeeding practices, and the timing of introduction of solid foods. In addition we collected a 3-day weighed food record at 18 months. Previously we reported associations between early dietary variables and BMI at 8 years, including positive associations between early intake of protein, meat and fruit at 18 months and measures of adiposity at 8 years [7]. In this paper we use a growth mixture model to empirically identify classes of BMI trajectories (from birth to 11.5 years) and examine the effects of breastfeeding, introduction of solids, as well as food and nutrient intake at 18 months on these BMI trajectories. 

## 2. Experimental Section

### 2.1. Participants

This study used data collected from birth to 11.5 years on subjects in CAPS. CAPS began as a randomized controlled trial (RCT) investigating the effects on the primary prevention of asthma of an intervention comprising house dust mite avoidance and omega-3 fatty acid supplementation from birth to five years [8]. Subjects who participated in CAPS were also studied at 8 and 11.5 years of age. Details on the study design, intervention, population, results from the RCT and the first 8 years of follow-up have been previously described [8,9,10]. In brief, pregnant women whose unborn children were at increased risk of developing asthma, because one or more parents or siblings had current asthma or wheezing, were recruited from antenatal clinics in western Sydney, Australia from 1997 to 2000. Exclusion criteria included babies from multiple births, gestational age less than 36 weeks, birth weight less than 2.5 kg, hospitalization for more than 1 week or serious illness, those with a pet cat at home and strict vegetarians. A total of 616 children were randomized at birth into active intervention or control groups. The study was approved by the Human Research Ethics Committees of the University of Sydney, Children’s Hospital at Westmead, and Sydney South West Area Health Services. 

We have previously shown that this cohort was similar in most respects to the population of western Sydney from which it was derived, but a higher proportion of both fathers and mothers of children in this cohort had tertiary education and were Australian-born, compared to those who did not participate in the study and the population of western Sydney in general [11]. The active supplement intervention comprised a fish-oil (omega-3) capsule, the contents of which were added to the child’s formula or food when the child ceased breastfeeding or by six months of age, whichever came first. Modification of the diet was minimal, requiring only the use of low omega-6 spreads and cooking oils in the preparation of the child’s food. The control group received a supplement of omega-6 oil, and omega-6 cooking oils and spreads. 

### 2.2. Measures

#### 2.2.1. Outcome Variable: Trajectories of BMI

Height and weight measurements were made by the study nurses at the following visits: 1, 3, 6, 9, 12, 18 months, and every 6 months thereafter until 5 years, and at 8 and 11.5 years. Weight and length records at birth were obtained from hospital birth records. Recumbent length was measured to the nearest 0.5 cm from birth to 2 years and standing height was measured at each visit from 2 years to 11.5 years (to the nearest 0.5 cm to 5 years and the nearest 1 cm thereafter). Weight was measured to the nearest 0.01 kg from birth to 2 years, nearest 0.1 kg from 2.5 to 5 years and nearest 1 kg at 8 and 11.5 years. Children were dressed in light clothing without shoes for the measurements. BMI was calculated using kg/m^2^. 

#### 2.2.2. Predictor variables

##### 2.2.2.1. Infant Feeding

Breastfeeding duration, among mothers who commenced breastfeeding at birth, was determined prospectively during the child’s first year by interview with mothers at 3-monthly intervals using standardized questions for monitoring breastfeeding [12]. Children were classified into three groups of breastfeeding duration: 0 to <3 months, ≥3 to <6 months and ≥6 months. Breastfeeding in this analysis refers to any breastfeeding. Information on water and juice consumption was not recorded in this study and therefore it was not possible to determine exclusive breastfeeding. Information about the introduction of breastmilk substitutes and solids was collected and used to determine rates of full breastfeeding which have been previously reported. [12] We note that of infants who were breastfed at one month of age 75% had not received any breastmilk substitutes or solids and similarly at three months of age 57% had not received any breastmilk substitutes or solids.

Early introduction of solids was determined by interview with mothers at 3 months using the question: “Has your baby ever been fed solid food?” 

##### 2.2.2.2. Dietary Intake around 18 Months

The diet was measured at the 18 month assessment in association with other medical assessments. Dietary intake of foods and nutrients was calculated from three-day weighed food records. A research dietitian instructed parents or caregivers to keep records on two weekdays and one weekend day, including methods for weighing and recording, and issued a food record booklet and set of Tanita digital kitchen scales. At the end of the recording period, the dietitian visited homes to collect records and check their completeness. Raw data from the food records were checked, coded and entered into a nutrient analysis program (Williams, Sydney, SERVE version 3.95, 1998) based on Australian Composition of Foods (National Food Authority, Canberra, NUTTAB 95 version 3.0, 1995) to derive estimates for food groups, energy and nutrient intake on average over the three days. Macronutrients were expressed in absolute amounts and as a percentage of total energy. For estimates of foods consumed and their contribution to total energy intake, food items were grouped into 16 food groups based on eight-digit codes and classifications used in the Australian National Nutrition Survey (NNS) [13]. In this analysis we have grouped foods as core foods and extra foods. Core foods were “dairy foods”, which were defined as milk and milk products, including yoghurt, cheese, ice-cream and custard; “milk”, which included skim, whole and evaporated milk; “fruit”; “vegetables”; “cereal foods”, which included bread, pasta, rice, breakfast cereals; and “meats”. Extra foods were cereal-based products (such as cookies and crackers), non-milk beverages (including juice, cordial, fruit drinks and soft drinks), fats and oils, snack foods, sugar, confectionery, savory sauces and condiments, fried potatoes, ice-cream and some miscellaneous foods [14]. 

##### 2.2.2.3. Parental Characteristics

Parental height and weight were assessed at the eight-year assessment and were measured by researchers, where possible, and otherwise by self-report. BMI was calculated, and mothers and fathers were classified as normal weight (BMI < 25 kg/m^2^), overweight (25 ≤ BMI < 30), or obese (BMI ≥ 30). Ethnicity was based on the child’s grandparents’ country of birth collected by questionnaire when the children were 4.5 years. Children were classified as belonging to an ethnic group (Caucasian, European, Middle Eastern, Indian, or Asian) if 3 or more of their grandparents were born in the same region; otherwise ethnicity was undefined. Information about maternal smoking during pregnancy, parents’ educational attainment and employment status before the child was born was obtained by interview at the perinatal visit. 

### 2.3. Statistical Analysis

We used a latent basis growth mixture model to define the classes of BMI trajectories, separately for boys and girls, using all available measurements of BMI from birth to 11.5 years using Mplus version 6.1 [15]. A latent basis growth mixture model [16] has been used by others to estimate trajectories of BMI [1]. It is a flexible growth curve model which allows the shape of the growth curve to take on nonlinear patterns by allowing basis coefficients to be estimated from the data. [17] We set the basis coefficient to 0 when time was equal to birth and set the basis coefficient to 1 when time was equal to 1 year. All remaining basis coefficients were allowed to be freely estimated from the data. When modeling the different classes we allowed the mean and basis coefficients (*i.e.*, the starting point and shape of the growth curve) to vary between the classes but the variances and covariances of the factors within the model were constrained to be equal across the classes. To determine the optimal number of classes for the BMI trajectories we ran the latent basis growth mixture model with 1 to 5 classes. All models were estimated using a maximum likelihood estimation with robust standard errors. We used the following criteria to determine the optimal number of classes (from 1 to 5): convergence, fit indices Bayesian Information Criterion (BIC); adjusted BIC; Akaike’s Information Criterion (AIC); entropy, class size, and interpretability [18]. 

Once the optimal number of classes was chosen, for boys and girls separately, we assigned each subject to the class for which they had the highest probability. Chi-squared tests were used to assess the relationship between breastfeeding, introducing solids early, parental characteristics and subsequent BMI class membership. Analysis of variance was used to assess the differences in mean macronutrient intake among BMI classes. Analysis of covariance was used to adjust the macronutrients expressed in units of 100 g of fat, carbohydrate, or protein respectively for total energy intake. Kruskal-Wallis tests were used to assess the difference of dietary intakes of food groups across BMI classes as intakes of food groups are not normally distributed. To assess the relationship between BMI classes and dietary intakes of food groups after adjusting for confounders, multinomial regression was used with food group intakes converted to quintiles. The response variable in this model was the BMI trajectory class membership. The following potential confounders were adjusted for: CAPS dietary intervention group (to incorporate the original study design), breastfeeding, parental obesity, ethnicity, smoking during pregnancy, and father’s education status. These potential confounders were included in this and our previous analysis because they have been associated with obesity in previous studies [7]. All statistical analyses assessing the relationships between dietary predictors and BMI classes were performed using SAS (version 9.2; SAS Institute Inc, Cary, NC).

## 3. Results

Of the 616 subjects in the original CAPS study, 370 (60%) subjects were included in this analysis as they were still participants in the study at 11.5 years. Half of the subjects were boys and half were girls. Complete data were available for all 370 subjects for breastfeeding and introduction to solids, and 18-month dietary data were available for 298 (81%) subjects. Of the 370 subjects, 255 (69%) had weight and height recorded at all 16 measurement occasions. The prevalence of overweight or obesity (≥85th percentile) [19] among boys at 3, 5, 8 and 11.5 years was 26%, 32%, 30% and 34%, respectively. The prevalence of overweight or obesity (≥85th percentile) [19] among girls at 3, 5, 8 and 11.5 years was 24%, 24%, 28% and 31%, respectively. 

Table 1 describes the characteristics of the subjects included in this analysis and compares them to those lost to follow-up at 11.5 years. Compared with those lost to follow-up, subjects included in this analysis had mothers and fathers who were older, more highly educated and more likely to be in full-time employment, a lower proportion of mothers who smoked during pregnancy, and higher rates of primigravida, but did not differ in other respects. 

**Table 1:** Comparison of characteristics of subjects included in this analysis and those lost to follow-up.

		Subjects included in this analysis (*N* = 370)	Subjects lost to follow up at 11.5 years (*N* = 246)	Difference between included and lost to follow-up
**Variable**		***n* (%)**	***n* (%)**	***P*-value ^1^**
Child’s gender				
	Male	187 (51)	125 (51)	0.95
	Female	183 (49)	121 (49)	
Maternal smoking in pregnancy				
	No	292 (79)	174 (71)	0.02
	Yes	78 (21)	72 (29)	
Age of parents at child’s birth (years, mean ± SD)				
	Mother	29.0 ± 5.1	27.6 ± 5.6	0.002
	Father	31.2 ± 5.9	30.2 ± 6.3	0.047
Australian born				
	Mother	272 (74)	185 (75)	0.64
	Father	255 (69)	166 (68)	0.71
Tertiary educated				
	Mother	191 (52)	85 (35)	<0.001
	Father	175 (47)	90 (37)	0.009
Full-time employment				
	Mother	183 (49)	95 (39)	0.008
	Father	323 (87)	195 (79)	0.008
Primigravida		131 (35)	68 (28)	0.04

^1^ The *P*-value to test the difference between those included and lost to follow-up in this study was calculated by a chi-squared test for all variables except age of the parents where a *t*-test was used.

Table 2 shows the model fit statistics for the growth mixture model with 1 to 5 classes. The 3-class model was chosen as the optimal class size for both boys and girls. The 4- and 5-class models resulted in some classes that included less than 10% of the sample and hence were excluded from further consideration. We chose the 3-class model because it had the lowest BIC, sample size adjusted BIC and AIC of the remaining models. 

**Table 2:** Growth mixture model fit statistics for 1 to 5 classes.

Sex	Number of classes	Parameters	AIC ^1^	BIC ^2^	aBIC ^3^	Entropy	LMR ^4^ *P*-Value
**Males**	**1**	20	10566	10631	10567	-	-
	**2**	37	9620	9740	9622	0.92	0.057
	**3**	54	9318	9492	9321	0.83	0.137
	**4**	71	9164	9394	9169	0.87	0.126
	**5**	88	9021	9305	9026	0.85	0.707
**Females**	**1**	20	9793	9857	9794	-	-
	**2**	37	9112	9231	9114	0.86	<0.001
	**3**	54	8976	9149	8978	0.85	0.442
	**4**	71	8892	9120	8895	0.81	0.353
	**5**	88	8830	9112	8834	0.79	0.731

^1^ Akaike’s Information Criterion; ^2^ Bayesian Information Criterion; ^3^ Sample size adjusted BIC; ^4^ Lo, Mendell and Rubin likelihood ratio test.

**Figure 1:**
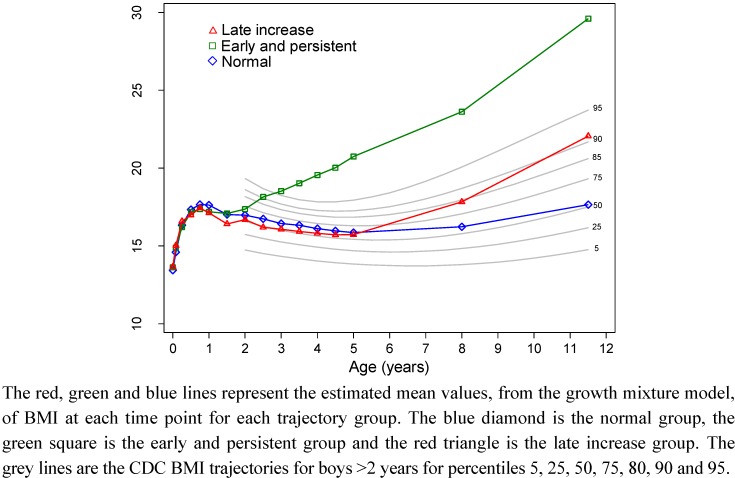
Boys BMI trajectory classes and the CDC BMI percentiles for boys.

Figure 1 shows the BMI trajectory classes for the 187 boys in relation to the Centers for Disease Control and Prevention (CDC) percentile curves for boys (which start from age 2 years) [19]. The three BMI trajectory classes can be described as: (1) normal BMI (*n* = 114, 61%), characterized by a growth curve which tracks along the 50th percentile; (2) early and persistent increase in BMI (*n* = 22, 12%), characterized by a BMI curve which is on the 75th percentile at 2 years and continues to increase and cross percentile curves to be above the 95th percentile at 11.5 years; (3) late increase in BMI (*n* = 51, 27%), characterized by 50th percentile tracking from birth to 5 years with subsequent increase to the 85th percentile at 8 years and the 90th percentile at 11.5 years. 

**Figure 2:**
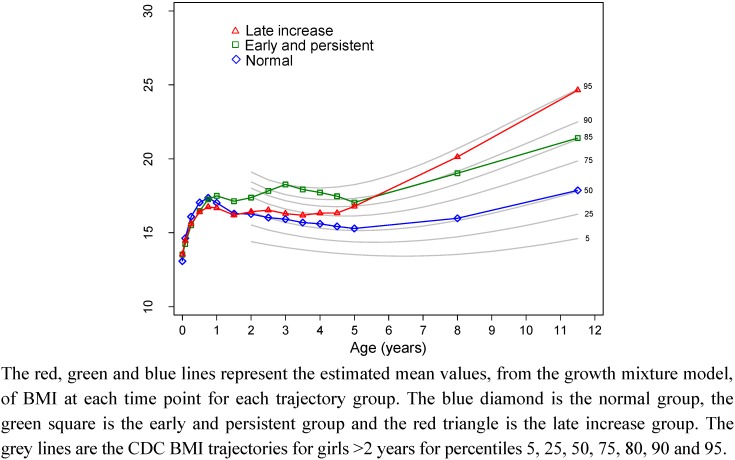
Girls BMI trajectory classes and the Centers for Disease Control and Prevention (CDC) BMI percentiles for girls.

Figure 2 shows the equivalent data for girls. The three BMI trajectory classes for the 183 girls can be described as: (1) normal BMI (*n* = 113, 62%), characterized by a growth curve which tracks along the 50th percentile; (2) early and persistently high BMI (*n* = 22, 12%), characterized by a BMI curve which increases to the 95th percentile at 3 years and only reduces slightly to be at the 85th percentile at 11.5 years; (3) late increase in BMI (*n* = 48, 26%), characterized by a curve which tracks on the 50th percentile from birth to 2 years at which time the BMI increases to the 85th percentile at 8 years and the 95th percentile at 11.5 years.

Of the 370 subjects who participated in the study at 11.5 years, 292 (79%) had a height and weight measurement at the 11.5 year assessment. The growth mixture modeling can accommodate missing data in the analysis. However, as a sensitivity analysis we ran the models with only those subjects with a height and weight recorded at 11.5 years (*n* = 292) and found similar class distributions and patterns for boys and girls. Specifically, all but two boys and all but six girls remained in the same class. 

We found no relationship between BMI trajectory class and breastfeeding or early introduction of solids (Table 3). We found significant associations between BMI trajectory class and maternal smoking, maternal education and maternal employment status in girls. Daughters of women who smoked heavily during pregnancy or were less educated or were not in full-time employment before the child was born were more likely to be in the late increase BMI group. Sons of women who were obese were more likely to be in the late increase BMI group. For both boys and girls, we found no relationship between BMI trajectory class and CAPS diet intervention group, overweight status of fathers, ethnicity, or father’s education.

The relationship between BMI trajectory class and macronutrient intake at 18 months is shown in Table 4. There was a significant association between BMI trajectory class and absolute intake of carbohydrates at 18 months in boys. Boys in the late increase BMI group had a larger mean intake of carbohydrate at 18 months than those in other classes (*P* = 0.04). This association was not significant after adjustment for total energy intake. There were no other significant relationships between BMI trajectory class and macronutrients or total energy intake in boys or girls. 

The relationships between BMI trajectories and intakes of food groups are shown in Table 5. In boys, there was a significant association between BMI trajectory class and intake of meat. Boys in the early and persistent BMI group had higher intakes of meat in absolute terms and as a percent of total energy intake than the other classes (*P* = 0.01 for both). Using multinomial regression to adjust for confounders the association remained significant (*P* = 0.03 and *P* = 0.01, respectively). After adjusting for confounders, the odds ratio comparing those in the early and persistent BMI class to the normal BMI class for one quintile increase in absolute meat intake in grams was 2.0 (95% CI: 1.2 to 3.4) and for one quintile increase in meat intake as a percentage of total energy intake was 2.3 (95% CI: 1.3 to 4.1).

**Table 3:** Relationship of BMI trajectory groups to dietary and non-dietary predictors, by sex.

		Boys BMI trajectory group										Girls BMI trajectory group							
		Overall	Normal		Early and persistent			Late BMI increase		*P* ^1^		Overall	Normal		Early and persistent		Late BMI increase		*P* ^1^
		*N*	*n*	(%)	*n*	(%)		*n*	(%)			*N*	*n*	(%)	*n*	(%)	*n*	(%)	
**Overall Sample**		**187**	**114**	**(61)**	**22**	**(12)**		**51**	**(27)**			**183**	**113**	**(62)**	**22**	**(12)**	**48**	**(26)**	
**Dietary predictors**																			
Breastfeeding duration																			
	0 to <3 months	73	45	(62)	8	(11)		20	(27)	0.27		75	49	(65)	8	(11)	18	(24)	0.25
	3 to <6 months	29	19	(66)	6	(21)		4	(14)			33	23	(70)	1	(3)	9	(27)	
	≥6 months	85	50	(59)	8	(9)		27	(32)			75	41	(55)	13	(17)	21	(28)	
Introduced solids by 3 months																			
	No	125	72	(58)	17	(14)		36	(29)	0.18		125	75	(60)	16	(13)	34	(27)	0.30
	Yes	42	30	(71)	2	(5)		10	(24)			35	26	(74)	3	(9)	6	(17)	
**Non-dietary predictors**																			
CAPS diet intervention group																			
	Control	88	52	(59)	10	(11)		26	(30)	0.81		95	60	(63)	9	(10)	26	(27)	0.54
	Active	99	62	(63)	12	(12)		25	(25)			88	53	(60)	13	(15)	22	(25)	
Parental weight status ^2^																			
	Both parents overweight or obese	63	36	(57)	6		(10)	21	(33)	0.19		73	45	(62)	10	(14)	18	(25)	0.68
	Only mother overweight or obese	29	13	(45)	4		(14)	12	(41)			20	11	(55)	4	(20)	5	(25)	
	Only father overweight or obese	45	27	(60)	6		(13)	12	(27)			54	33	(61)	4	(7)	17	(31)	
	Both normal	21	16	(76)	2		(10)	3	(14)			11	9	(82)	0	(0)	2	(18)	
	Undefined	29	22	(76)	4		(14)	3	(10)			25	15	(60)	4	(16)	6	(24)	
Fathers BMI																			
	Normal	45	28	(62)	5		(11)	12	(27)	0.19		24	16	(67)	3	(13)	5	(21)	0.95
	Overweight	64	41	(64)	3		(5)	20	(31)			77	46	(60)	10	(13)	21	(27)	
	Obese	44	22	(50)	9		(20)	13	(30)			50	32	(64)	4	(8)	14	(28)	
	Missing/refused	34	23	(68)	5		(15)	6	(18)			32	19	(59)	5	(16)	8	(25)	
Mothers BMI																			
	Normal	67	46	(69)	7		(10)	14	(21)	0.045		67	42	(63)	5	(7)	20	(30)	0.48
	Overweight	45	29	(64)	4		(9)	12	(27)			51	34	(67)	8	(16)	9	(18)	
	Obese	47	20	(43)	6		(13)	21	(45)			42	22	(52)	6	(14)	14	(33)	
	Missing/refused	28	19	(68)	5		(18)	4	(14)			23	15	(65)	3	(13)	5	(22)	
Child's grandparents ethnicity/country of birth ^3^																			
	Caucasian	100	62	(62)	8		(8)	30	(30)	0.36		98	60	(61)	12	(12)	26	(27)	0.86
	European	17	10	(59)	4		(24)	3	(18)			18	11	(61)	1	(6)	6	(33)	
	Middle Eastern/ Indian/Asian	19	9	(47)	4		(21)	6	(32)			24	17	(71)	2	(8)	5	(21)	
	Undefined	51	33	(65)	6		(12)	12	(24)			43	25	(58)	7	(16)	11	(26)	
Cigarettes during pregnancy																			
	None	146	90	(62)	15		(10)	41	(28)	0.53		146	96	(66)	15	(10)	35	(24)	0.04
	1–10/day	28	16	(57)	6		(21)	6	(21)			22	13	(59)	4	(18)	5	(23)	
	11–40/day	13	8	(62)	1		(8)	4	(31)			15	4	(27)	3	(20)	8	(53)	
Father’s Education																			
	≤10 years of school	63	33	(52)	9		(14)	21	(33)	0.20		57	32	(56)	7	(12)	18	(32)	0.13
	11–12 years of school	37	27	(73)	5		(14)	5	(14)			34	18	(53)	8	(24)	8	(24)	
	Tertiary Education	85	54	(64)	8		(9)	23	(27)			90	61	(68)	7	(8)	22	(24)	
Mother’s Education																			
	≤10 years of school	53	30	(57)	6		(11)	17	(32)	0.72		57	38	(67)	3	(5)	16	(28)	0.047
	11–12 years of school	34	24	(71)	3		(9)	7	(21)			35	15	(43)	8	(23)	12	(34)	
	Tertiary Education	100	60	(60)	13		(13)	27	(27)			91	60	(66)	11	(12)	20	(22)	
Father worked full time before baby born																			
	No	22	12	(55)	4		(18)	6	(27)	0.59		25	17	(68)	2	(8)	6	(24)	0.73
	Yes	165	102	(62)	18		(11)	45	(27)			158	96	(61)	20	(13)	42	(27)	
Mother worked full time before baby born																			
	No	90	56	(62)	7		(8)	27	(30)	0.24		97	57	(59)	7	(7)	33	(34)	0.01
	Yes	97	58	(60)	15		(15)	24	(25)			86	56	(65)	15	(17)	15	(17)	

^1^ *P*-value is from a chi-squared test; ^2^ Obese was defined as BMI ≥ 30, overweight: BMI ≥ 25 to <30, Normal weight: BMI < 25; ^3^ Ethnicity was defined based on the child’s grandparent’s country of birth. An ethnic classification was made if 3 or more grandparents were from the same country of birth region; otherwise ethnicity was undefined.

**Table 4:** Total energy and macronutrient intakes at 18 months by BMI trajectory group and sex.

		Boys BMI trajectory group									Girls BMI trajectory group							
		Normal( *n* = 98)		Early and persistent( *n* = 16)		Late BMI increase( *n* = 41)		*P*-value			Normal( *n* = 92)		Early and persistent ( *n* = 18)		Late BMI increase( *n* = 33)		*P*-value	
Variable	Unit	Mean	SD	Mean	SD	Mean	SD	Unadjusted ^1^	Adjusted ^2^		Mean	SD	Mean	SD	Mean	SD	Unadjusted ^1^	Adjusted ^2^
Total energy	MJ	4.35	0.83	4.32	1.15	4.68	1.14	0.16			4.25	0.93	4.41	0.96	4.13	1.32	0.66	
Protein	100 g of protein	0.40	0.11	0.42	0.11	0.43	0.12	0.27	0.27		0.39	0.11	0.40	0.09	0.38	0.13	0.82	0.87
Percentage of total energy from protein	%	15.46	2.65	16.78	2.99	15.52	2.51	0.18			15.50	3.02	15.57	2.44	15.84	2.51	0.84	
Fat	100 g of fat	0.43	0.12	0.44	0.13	0.45	0.15	0.77	0.15		0.41	0.11	0.43	0.10	0.42	0.14	0.91	0.58
Percentage of total energy from fat	%	36.47	5.76	37.32	4.58	34.91	5.07	0.21			35.88	5.82	35.86	5.64	37.48	5.45	0.37	
Carbohydrates	100 g of carbohydrates	1.26	0.27	1.22	0.37	1.40	0.33	0.04	0.19		1.26	0.35	1.30	0.37	1.19	0.44	0.51	0.69
Percentage of total energy from Carbohydrates	%	49.72	7.51	47.65	6.30	51.26	6.55	0.21			50.30	7.77	50.22	7.33	48.49	6.81	0.49	

^1^ From analysis of variance; ^2^ Adjusted for total energy intake by analysis of covariance.

**Table 5:** Food group intake at 18 months by BMI trajectory group and sex.

		Boys BMI trajectory group				Girls BMI trajectory group				
		Normal( *n* = 98)	Early and persistent( *n* = 16)	Late BMI increase( *n* = 41)			Normal( *n *= 92)	Early and persistent( *n* = 18)	Late BMI increase( *n* = 33)	
Food group	Units	Median	Median	Median	*P*-value ^1^		Median	Median	Median	*P*-value ^1^
**Core Foods**										
Dairy foods ^2^	g	554.3	433.1	527.2	0.24		473.1	576.5	480.9	0.45
	% of total energy	38.1	33.4	35.9	0.56		37.0	40.6	32.7	0.28
Milk ^3^	g	478.8	401.1	407.0	0.46		398.7	510.0	380.8	0.74
	% of total energy	29.4	24.8	23.5	0.53		28.0	26.7	25.3	0.85
Fruit	g	57.3	78.0	79.7	0.20		60.7	61.3	57.7	0.75
	% of total energy	3.8	4.0	5.0	0.52		3.6	5.4	4.2	0.73
Vegetables	g	43.2	57.3	36.3	0.49		35.3	43.3	43.0	0.48
	% of total energy	3.6	3.4	2.8	0.64		3.8	3.6	3.8	0.97
Cereals ^4^	g	60.7	52.1	63.4	0.79		54.2	59.9	58.3	0.87
	% of total energy	13.3	14.4	14.1	0.94		14.0	13.4	14.1	0.86
Meats	g	18.6	38.8	24.1	0.01		26.4	25.4	35.0	0.57
	% of total energy	4.4	9.2	4.1	0.01		5.6	4.9	7.3	0.38
**Extra Foods**										
Total extra foods ^5^	g	118.3	131.3	124.3	0.81		113.7	112.4	82.5	0.60
	% of total energy	24.0	26.2	24.5	1.00		24.8	25.1	24.8	0.99
Non-milk beverages ^6^	g	128.3	148.3	150.9	0.54		133.3	139.2	100.0	0.50
	% of total energy	6.2	8.5	6.4	0.70		7.2	5.9	5.6	0.27
Sweetened drinks ^7^	g	15.0	31.7	17.8	0.93		21.7	7.5	1.0	0.23
	% of total energy	2.1	4.0	2.1	0.41		3.4	1.2	2.1	0.26
Fried potatoes	g	3.3	0.0	3.3	0.73		5.2	1.7	1.3	0.67
	% of total energy	0.9	0.0	0.7	0.76		1.4	0.5	0.3	0.73
Salty snacks ^8^	g	0.0	0.0	0.0	0.98		0.0	0.0	0.0	0.76
	% of total energy	0.0	0.0	0.0	0.98		0.0	0.0	0.0	0.79
Confectionery	g	1.3	3.3	4.0	0.41		4.0	3.8	3.3	0.99
	% of total energy	0.4	1.6	1.0	0.47		1.7	1.9	1.9	0.99
Cereal based foods ^9^	g	18.0	11.0	26.7	0.15		22.5	28.5	18.5	0.56
	% of total energy	7.1	4.6	8.6	0.19		9.1	8.4	7.0	0.59

^1^ Estimates were made using a Kruskal-Wallis test; ^2^ Milk and milk products including milk, yoghurt, cheese, ice-cream, custard; ^3^ Milk including skim, whole and evaporated milk; ^4 ^Includes bread, breakfast cereal, pasta, rice, *etc.*; ^5^ Includes cereal-based products (such as cookies, *etc.* see footnote 9), non-milk beverages (except for fruit juice), fats and oils, snack foods, sugar, confectionery (chocolate, jellies, energy bars), savory sauces and condiments, miscellaneous foods, fried potatoes, and ice-cream; ^6^ Juice, cordial, fruit drinks and soft drinks; ^7^ Cordial, fruit drinks, and soft drinks; ^8^ Potato crisps, cheese snacks, corn chips; ^9^ Cookies, crackers, doughnuts, cakes, pies, buns, muffins, pizza, *etc*.

## 4. Discussion

We have identified three BMI growth trajectories between birth and age 11.5 years, which qualitatively differ between boys and girls. We have shown that neither breastfeeding nor early introduction of solids influenced the subsequent trajectory of BMI to 11.5 years. In boys, however, higher intake of meat (both absolute and relative to energy consumption) at 18 months was associated with a BMI trajectory characterized by early and sustained high BMI. Similar to the findings in this and our previous study [7], others have shown an association between meat intake and obesity in children (males) [20,21] and adults [22]. Although we did not find an association with fat or energy intake, we previously reported that the meats consumed by our sample were high in fat and calories; chicken nuggets, ground beef, beef sausages and ham were the most commonly consumed [23]. Further, we found that those in the highest quintile of meat intakes had higher energy and fat intakes and weighed more than other quintile groups at age 18 months, for boys and girls combined [23]. 

Boys who had a higher intake of carbohydrate (in absolute terms but not relative to energy consumption) at 18 months had a BMI trajectory characterized by a late increase in BMI. Several authors have found no association or a negative association between obesity and total and refined carbohydrate intake [24], with the exception of sugar sweetened beverages, although some mixed findings, preponderance of evidence points to sugar sweetened beverages as contributing to child obesity [25]. We previously reported the highest sources of carbohydrate in our sample of 18 month old children: breads and cereals (24%), milk and dairy foods (21%) and sugar sweetened beverages (17%) which provided the largest source of refined sugar in the diet of these children [26]. Intake of carbohydrate and refined sugars or sugar sweetened beverages in early childhood may track to later childhood or be a marker for later high consumption of energy dense foods. Neither macronutrients nor food group intakes at 18 months were associated with BMI trajectory class in girls. It is unclear why no associations were seen among girls for early diet, but it is possible that the socio-economic confounders we identified for girls and not boys may have limited our ability to detect any associations. 

The BMI trajectory classes for boys and girls had some similarities. The majority of both boys and girls were members of a class that followed the 50^th^ percentile from birth to 11.5 years. The remaining classes in boys were one class that exhibited an increasing and persistently high BMI class from around 2.5 years, and another class that roughly followed the 50th percentile to around 5 years at which time BMI increased to the 90th percentile at 11.5 years. In girls the remaining classes were one class with persistently high BMI that started around 2 years and increased to the 95th percentile at 3 years and remained high but reduced to around the 85th percentile, and another class that started to increase to the 75th percentile at 4 years and continued to increase to the 95th percentile at 11.5 years. Our finding that a 3-class solution is the optimal fit for this data is similar to other studies which have found a 3-class solution fits weight developmental trajectories [2,5,6]. Our class descriptions are also similar to these studies which all share a normal BMI/weight group that is the largest group of the sample, an early onset of obesity/always overweight group and a late onset or gradually becoming overweight group [2,5,6]. Our class descriptions for girls are also similar to three of the four groups identified by Ventura *et al.* [1].

The absence of any association between BMI trajectories and breastfeeding is consistent with Pryor *et al*. who examined BMI trajectories from 5 months to 8 years and found no relationship [5]. These results are in contrast to others who have found longer duration of breastfeeding reduces the risk of being in groups characterized by early onset of overweight [1,2] and other research that has found breastfeeding is protective against later obesity [27,28,29].

Our results showing a relationship between markers of low socioeconomic status (smoking in pregnancy [30], low levels of maternal education and lack of full time employment) and BMI trajectory classes characterized by high BMI in girls are similar to those observed by Balistreri [6], who also found a gender difference in the relationship between socioeconomic status and weight groups. In that study, low parental income and parental education were risk factors for girls but only low parental education was a risk factor for boys. Our finding that girls whose mother smoked during pregnancy were at greater risk of being in the class with early and persistently increasing BMI is consistent with the results of Pryor *et al.* [5]. 

The strengths of this study were several: the birth cohort study design; the rigorous methods used to measure infant feeding and early diet, including quarterly measurements of breastfeeding throughout the first year, as opposed to recalled duration measured one or more years later, and 3-day weighed record at 18 months to quantify food and nutrient intakes; and repeated measures of heights and weights throughout childhood. Participants in the original RCT were not a random sample of the newborn population, and although their selection for risk of asthma and atopy would be unlikely to affect weight and diet outcomes, the exclusion of low birth weight infants may have underestimated obesity and influenced our findings regarding weight trajectories.

The timing of our measures of early diet, during a period of rapid transition from a milk-based to solid food diet may not be reflective of the habitual solid food diet that follows after this period, and we did not have dietary measures between the 18 month and 8 year assessments. We also had a loss to follow-up over the 11.5 years. Although the loss was not large, potential bias cannot be ruled out, because those lost to follow-up were of lower socio-economic status, a factor related to BMI status among girls. It is also possible that the number of subjects in this analysis was too small to detect important and significant differences in some variables. The difficulty that we and other researchers face in finding associations between putative dietary risk factors and childhood obesity is not surprising, for a variety of reasons, among them the relatively small energy imbalance that leads to overweight and obesity and has led to the obesity epidemic [31].

An additional strength of this study was the use of the latent basis growth mixture model. This model allows the complex and different shapes of BMI growth over time to be estimated by the model and does not force a polynomial shape to the data. Due to the sample size in this study our capacity to detect more classes may have been limited. We were unable to detect classes that exhibit an underweight growth pattern as shown by Huang *et al.* [4]. 

## 5. Conclusions

Breastfeeding duration less than six months and the early introduction of solids did not adversely influence BMI trajectories in our sample but high intakes of meat, particularly high fat varieties, and high intakes of carbohydrate at age around 18 months were associated with a high BMI trajectory in boys. It is not clear whether these dietary factors confer a direct risk of higher BMI in childhood or are markers for other dietary patterns that are present early and/or develop through childhood and contribute to higher BMI. 
